# Etiologic Spectrum of Intestinal Obstruction in Ningxia District: A Retrospective Analysis of 4908 Cases in a 10-Year Period

**DOI:** 10.1155/2019/4935947

**Published:** 2019-06-26

**Authors:** Wei Jiang, Wenyan Li, Qian Hao, Yuping Yao, Yajun Li, Jun Ge, Huihong Zhai

**Affiliations:** ^1^Department of Gastroenterology, Beijing Friendship Hospital, Capital Medical University, Beijing 100050, China; ^2^Beijing Key Laboratory for Precancerous Lesion of Digestive Diseases, Beijing 100050, China; ^3^National Clinical Research Center for Digestive Diseases, Beijing 100050, China; ^4^Department of Digestive Diseases, General Hospital of Ningxia Medical University, Yinchuan 750004, China; ^5^Ningxia Medical University, Yinchuan, 750004 Ningxia Hui Autonomous Region, China

## Abstract

**Background:**

Economic disparity contributes to the variation of intestinal obstruction (IO) etiologic spectrum. Clarifying the etiology distribution in local regions can help to unravel IO and promote early diagnosis, henceforth making sure standardized therapeutic interventions.

**Methods:**

Medical data of 4908 inpatients diagnosed with IO admitted to the General Hospital of Ningxia Medical University between January 2004 and December 2013 were recruited and analyzed retrospectively. The associated profiles included demographic features, clinical manifestations, and previous therapeutic operations.

**Results:**

4908 cases of intestinal obstruction were identified during the period of study. It denoted that the hospitalization rate of IO has maintained upward momentum; the top four causes of IO were adhesion, tumor, intussusception, and hernias. These covered up nearly 80% of the total constitution. Among them, adhesive intestinal obstruction accounted for 45.17%, malignant bowel obstruction for 21.09%, intussusception for 8.72%, and hernia for 4.73%; abdominal surgery constituted for the majority (78.62%) of adhesive obstruction. The followed up analysis also found that appendectomy accounted for the biggest percentage, 28% of operation cases. Malignant bowel obstruction can have a rate of 96.43% in 1035 cases led by tumor lesions. Of which, the primary intestinal malignant tumor accounted for 68.64% and metastatic tumors for 31.36%. Nearly 50% occurred in the large intestine. The overall mortality of all 4908 cases was 4.7%.

**Conclusion:**

The hospitalizations of IO delineated an increasing trend. Adhesion was the main etiology in IO. The odds of malignant bowel obstruction was increasing in the proportion of IO. There were some differences towards the etiologic spectrum compared with western countries.

## 1. Introduction

Intestinal obstruction (IO) has been one of the most common underlying etiologies of acute abdomen since 1900s [1, 2]. According to a retrospective analysis performed in the National Hospital of Zinder, IO can account for 27.94% in surgery hospital admissions [3]. In resource-limited countries such as Nigeria, which has grossly inadequate facilities and manpower, IO was associated with high mortality and poor response to treatment [4]. Since the latest two decades, the morbidity and mortality of IO have not changed whether in western countries or in a less-developed area [5].

Many regions and countries have denoted different tendency towards etiologic spectrum of IO. In the past few years, adhesive intestinal obstruction and malignant bowel obstruction have replaced hernia, the dominant cause in early stage [6], shifted to the most common cause of IO in western countries [7]. However, in undeveloped countries, hernia is still the most common etiology of IO and postoperative adhesions increase annually [8]. The exact cause of obstruction can influence the prognosis of patient; henceforth, a study needs to be investigated to clarify the trend towards IO etiology spectrum and make diagnosis more specifically to certain patients, leading to a better prognosis.

In the past several decades, the etiologic spectrum of IO has changed significantly in China [9]. However, China has a great piece of land with large population. Regional economic development is unbalanced among various districts. The purpose of the study is to analyze the profiles of patients suffering from IO in Ningxia, one of the most relatively backward financial regions. We made a retrospective analysis of patients diagnosed with IO from Jan 2003 to Dec 2013. Current epidemiology of IO could be investigated and highlighted in Ningxia district. Valid data could be obtained towards the local etiological spectrum and referred by physicians on prevention and treatment. Henceforth, it can raise suspicion index and reduced delays in diagnosis and intervention options so that improved prognosis of patients could be achieved.

## 2. Patients and Methods

### 2.1. Participant Selection

**Figure 1:**
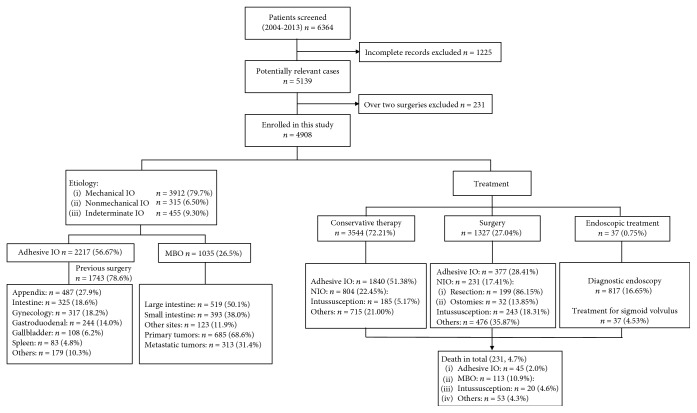
Flow chart of the study.

This was a retrospective, observational study carried out at the General Hospital of Ningxia Medical University from January 2004 to December 2013. Once admitted into hospital, profiles of patients on demography (age, gender, and etiology), symptoms, previous history of surgery, physical examination, and X-rays would be obtained by physicians in emergency room and stored in electronic hospital database. Based on the data collected and in terms of profiles towards presenting symptoms, physical examination, and imaging studies such as plain abdominal radiographs or later on operations if necessary, a diagnosis of IO would be confirmed followed by conservative therapy or urgent surgery. The malignancy would be made clear based on pathology whether by diagnostic endoscopy or during operation. The data management staff extracted data from database according to the conditions suggested by researchers in our study. There are patients who had undergone over two types of surgery (*n* = 231); incomplete records (*n* = 1225) were excluded from our study (Figure 1).

This study was approved by the ethics committee of the General Hospital of Ningxia Medical University.

### 2.2. Definition

Mechanical intestinal obstruction (MIO) was defined as conditions that occurs in case of failure of propagation of intestinal contents, associated with intra-abdominal hypertension and abdominal compartment syndrome. Nonmechanical intestinal obstruction (NMIO) refers to that the symptoms of IO that occurred due to the dysfunction of enteric nerve and smooth muscle or the contraction weakness, including dynamic intestinal obstruction, acute enteric vascular ischemia. We differentiated the socioeconomic status in terms of work types. As the economic status was negatively correlated with the physical requirement, mild class, moderate class, and severe class were categorized to stand for mental workers, intermediate workers, and manual workers [10]. Complete intestinal obstruction is an intestinal obstruction in which the intestinal tract is completely blocked, resulting in intestinal contents not being able to pass. Incomplete bowel obstruction is a type of condition that part of the intestinal lumen was occluded through the obstruction point where a small amount of gas and fluid can get through. The clinical manifestations are closely similar to the complete intestinal obstruction but in a lesser degree. Postoperative adhesion is defined as biochemical and cellular response that happens to repair the peritoneum after operative trauma, resulting in intestinal contents to not pass smoothly. Malignant bowel obstruction is referred to as the terminal complication of patients for advanced malignancy, especially colorectal and ovarian cancers, but can also be seen in other abdominal and nonabdominal malignancies. It often manifests the symptoms of intestinal obstruction which necessitates the multidisciplinary palliative therapy [11].

### 2.3. Statistical Analysis

All associated profiles were collected onto standard forms and then stored in the electronic hospital database. All statistical analysis was performed using SPSS 17 (SPSS Inc., Chicago, IL, USA) statistical software for Windows. We analyzed the statistical data with the comparison of proportion test.

## 3. Results

### 3.1. Prevalence of Intestinal Obstruction

**Figure 2:**
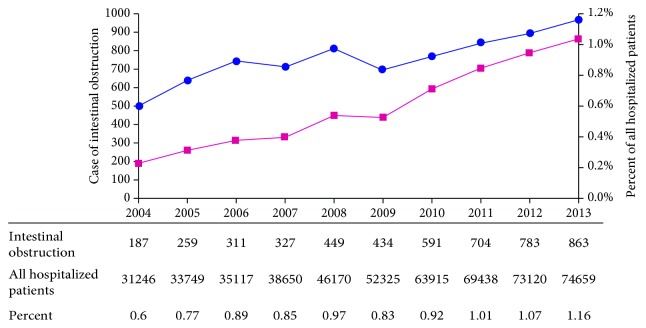
The proportional trend chart of intestinal obstruction.

From January 2004 to December 2013, 4908 patients were diagnosed with intestinal obstruction in the General Hospital of Ningxia Medical University. 187 patients of IO were admitted into hospital which took a rate of 0.60% of all hospitalizations in 2004 while in 2013 the number can reach to 863 (1.16%). It showed an upward trend over these years (Figure 2).

### 3.2. Demographic Characteristics

Among 4908 cases of IO, males accounted for 60% (*n* = 2945); pediatric patients (less than 18 years old) accounts for 20.50% (*n* = 1006, year ± SD: 4.8 ± 4.4). Adult patients (between 18 and 50 years old) covered a total of 1558 cases (31.74%, year ± SD: 37.9 ± 8.8), while elders (larger than 50 years old) of 2344 cases (47.76%, year ± SD: 65.9 ± 9.2). The majority of them come from urban area (60.68%, *n* = 2978) and the remainder in rural area (39.32%, *n* = 1930). The most common type of socioeconomic status lies in a severe class (59.29%, *n* = 2910), followed by a moderate class (16.71%, *n* = 820) and a mild class (24.00%, *n* = 1178) (Table 1).

All cases have undergone X-ray examination, and 2049 patients made diagnosis based on CT scan. 817 subjects underwent diagnostic endoscopy. Of these patients, 4138 subjects were classified into mechanical IO (84.20%) and nonmechanical IO recruited 315 cases (6.50%). There were also 455 cases (9.30%) of complaint of the symptoms of intestinal obstruction, and X-ray or other modalities can approve the existence of such disorder. However, the etiology remains unclear combining with all examinations. We sorted them out and allocated them into “indeterminate IO” (Table 1, Figure 1). The obstruction site was most common in the small intestine which was 3072 (62.59%) followed by the large intestine, 1330 (27.10%), and the indeterminate site, 505 (10.31%) (Table 1, Figure 1). As to the indeterminate sites, part of the patients can be diagnosed with intestinal obstruction combing with clinical manifestations, abdominal X-ray, or CT scanner. Malignancy can be found according to the clinical features and biopsy sent for pathology. However, inadequate evidence resulting from the lack of further treatment may confuse the physicians whether the malignancy occurred in the colon or small intestine. Towards the degree of obstruction, the quantity of incomplete intestinal obstruction was 2831 (57.68%), while complete intestinal obstruction was 2077 (38.28%) (Table 1). The length of hospital stay counts from 1 to 157 days (13.34 ± 12.79).

### 3.3. Etiology of Intestinal Obstruction

**Figure 3:**
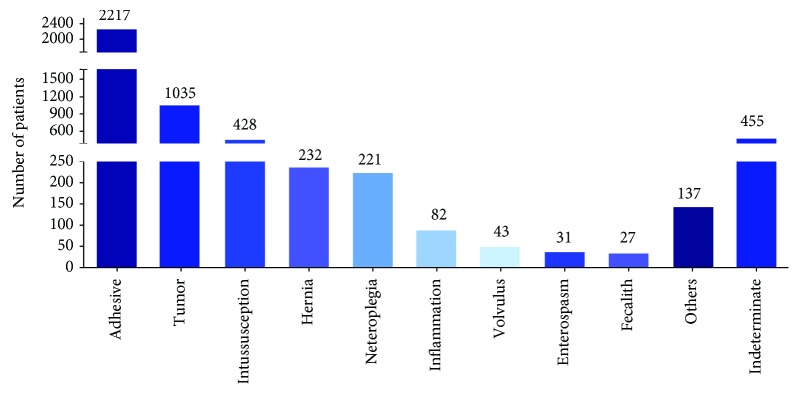
Causes of intestinal obstruction.

The most common four types of IO were as follows: adhesion, malignant bowel obstruction, intussusception, and hernia. The number of cases of intestinal obstruction caused by these four etiologies closes to 80% of the total. Among them, adhesive intestinal obstruction was 45.17%, tumor obstruction was 21.09%, intussusception was 8.72%, and hernia was 4.73% (Figures 1 and 3).

**Figure 4:**
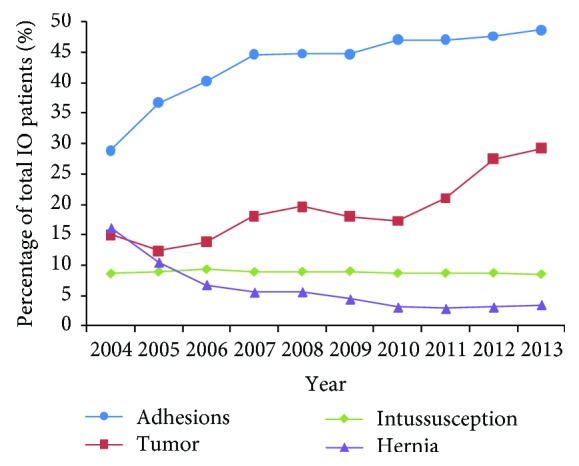
The change trend of etiology spectrum of intestinal obstruction.

From 2004 to 2013, adhesive intestinal obstruction and malignant bowel obstruction increased significantly while IO caused by hernias decreased rapidly. The ratio of intussusception to overall etiology remained stable (Figure 4).

### 3.4. Etiology Analysis of Adhesive Intestinal Obstruction

**Figure 5:**
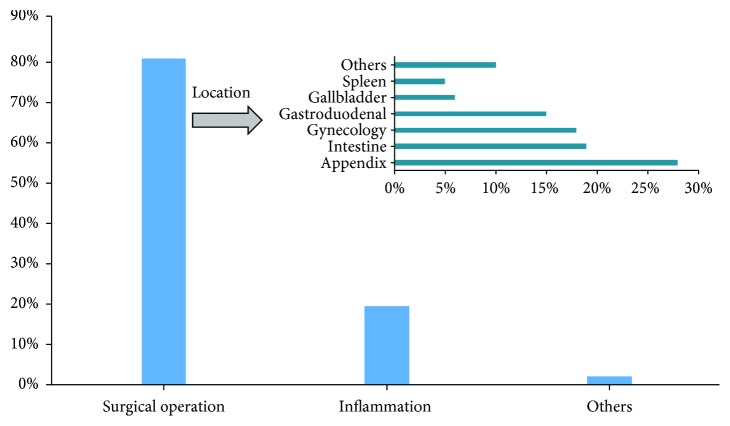
Etiological analysis of adhesive intestinal obstruction.

Among the patients with adhesions IO, previous history of abdominal surgery accounted for 78.62% (1743/2217), adhesions caused by intra-abdominal inflammation took for 19.17% (425/2217), and other factors made up of 2.21% (49/2217). The classification of abdominal surgery was categorized that appendectomy is the most common, accounting for 28% (487/1743), followed by intestinal surgery for 19% (325/1743) and gynecological surgery for 18% (317/1743) (Figures 1 and 5).

### 3.5. Etiology Analysis of Neoplastic Intestinal Obstruction

**Figure 6:**
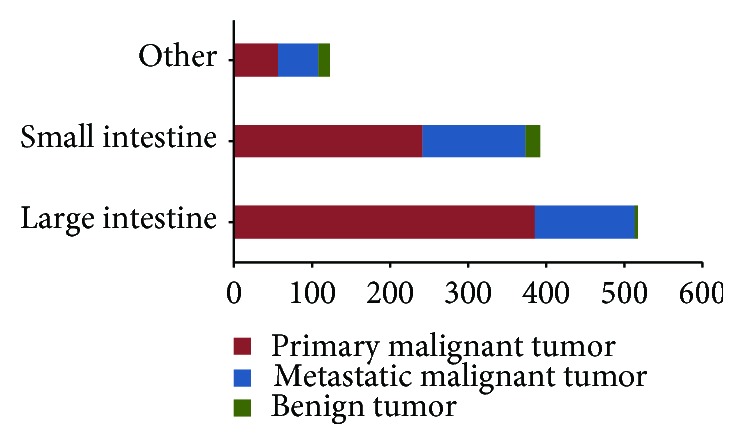
Types of analysis of intestinal obstruction that caused by tumor.

Tumor lesions led to 1035 cases of intestinal obstruction that 96.43% (998/1035) were malignant and 3.57% (37/1035) were benign tumors. In malignant tumors, primary malignant tumors accounted for 68.64% (685/998), and metastatic tumors made up of 31.36% (313/998). As for the type of tumors, 50% (519/1035) occurred in the large intestine, 38% (393/1035) in the small intestine, and 12% (123/1035) in the indeterminate sites (Figures 1 and 6).

### 3.6. Treatment and Prognosis

A total of 3544 patients (72.21%) underwent conservative therapy, and 1327 cases for surgery (27.04%). In addition, 37 subjects (0.75%) of 817 cases under diagnostic endoscopy were treated due to sigmoid volvulus (Figure 1). As to the conservational therapy, according to the category of etiology, adhesive intestinal obstruction took the highest proportion of conservative therapy (1840/3581, 51.38%) while the intussusception remained the lowest (185/3581, 5.17%). The conservative therapy for neoplasm cases and others accounted for 804/3581 (22.45%) and 752/3581 (21.00%), respectively. While the surgery covered up 1327 cases (27.04%), of these, other etiology consisting of hernia, Meckel's diverticulum, duodenum ulcer, etc. takes the majority part (*n* = 476, 35.87%), then followed by adhesive IO (*n* = 377, 28.41%), neoplastic intestinal obstruction (*n* = 231, 17.41%), and intussusception (*n* = 243, 18.31%), respectively. Among the neoplastic IO group, the surgical treatment options included resection and ostomies. The number for malignant bowel obstruction was 137 (81.07%) and 32 (18.93%), respectively (Figure 1). Of the 32 cases of ostomies, 26 subjects (81.25%) underwent temporary ostomies to relieve symptoms while 6 cases (18.75%) for definitive ostomies.

The overall mortality of all 4908 cases was 4.7%. The mortality of conservative treatment for intestinal obstruction was 4.4%, while the figure can reach 5.7% for surgical operation. As to the neoplasm patients, the mortality was 10.8% which is the highest among all groups in conservative treatment, while in the intussusception group, the mortality can reach 1.6%, which is the lowest (Figure 1).

## 4. Discussion

Over the recent years, IO has become one of the leading life-threatening emergencies [7]. The etiology spectrum for IO is closely connected with economy and geographic distribution. The aim of this study was to discuss and clarify the etiologic spectrum in the west of China, the undeveloped region. It showed in our study that the hospitalization rate of IO was in an increasing trend by years. The two main causes for IO were adhesions and tumors, and the proportion of these is increasing by year, while as for the hernia, the ratio has been lowered. Appendectomy was the most frequent intervention causing adhesive intestinal obstruction. Primary malignant tumor sited in the large intestine constituted for the most cases in malignant obstruction.

As the socioeconomic and medical insurance increased, variation exists in the etiology of intestinal obstruction in terms of geographic distribution. In western countries, the main constitution of IO is adhesive obstruction and gastrointestinal tumors [12], while in some developing even poor countries, inguinal hernia still takes up the majority of the etiology [13]. The study showed that intestinal obstruction caused by adhesions and tumors increased in Ningxia during a 10-year period, while hernias causing intestinal obstruction decreased by year. The most common etiology in an early stage (1930s-1980s) in China was inguinal hernias [6]. However, the economy has developed rapidly in the past 3 decades, even in the financially backward western region. In our study, it showed that during the 10-year period in certain district, the etiologic spectrum depicts a changing trend as the local economy develops.

Our study uncovered that among patients of adhesive obstruction, abdominal surgery accounted for majority. Further analysis of the history of abdominal surgery denoted that appendectomy was the most common intervention to cause adhesive intestinal obstruction, followed by intestinal surgery and gynecological surgery. A population-based study performed by Barmparas et al. showed that the highest incidence of intestine obstruction after laparotomy was ileal pouch-anal anastomosis, followed by open colectomy, which is in contrast with the results in our study [14]. Appendicitis is one of the acute abdomens with high incidence. Previous studies reported that 10.7% IO occurred after open surgery during 64 months while 1.3% obstruction happened after laparoscopic appendectomy for 4-6 years [3]. We mainly analyzed the patients' profiles from 2004 to 2013 when laparoscopy cases were not routinely recruited in our hospital. It may prompt us that the ratio of adhesive obstruction would decrease with the application of laparoscopy.

Among patients with malignant obstruction, primary bowel malignant tumors made up for majority. Colorectal cancer is the third most common malignancy in the developed world, and about 25-40% patients present with intestinal obstruction or perforation [15]. It has been reported in China that an upward trend in incidence of colorectal cancer occurred during recent decades. According to statistics, the annual report of China's cancer in 2015 showed that the incidence and the mortality rate of colorectal cancer ranked fifth in categories of tumors [16]. In our study, we could also find that the incidence of bowel tumors rose by years in IO patients. Once it progressed to relatively highly malignant, the symptoms of IO would occur as the acute abdomen. Tuca et al. reported that it accounted for the highest percentage of malignant bowel obstruction and approximately 3% to 15% of all IO hospitalizations [17]. In our study, 18.58% of IO patients were diagnosed with malignant bowel obstruction. Meanwhile, there were senior predominances of patients taking up in this proportion of etiology. The annually increasing data denoted that to lower the incidence of IO, more attention should be paid on malignant tumors.

The main limitation of our study is that patients in wide range of ages were enrolled in data profiles. The most common etiology of intestinal obstruction in pediatric patients was intussusception, so that the distribution of the etiology would be influenced. Another limitation lies that this study confirmed that postoperative adhesion was the primary cause of adhesive intestinal obstruction, but detailed profiles of the surgical methods such as open surgery, laparoscopy, preoperative perforation, and other factors affecting adhesion formation have not been analyzed in detail. Lastly, our study was limited within one province in China; it may not reflect the epidemiological spectrum in other regions and countries.

## 5. Conclusion

There was a rising incidence of patients admitted into hospital due to intestinal obstruction in Ningxia district, in nearly 10-year period. Postoperative adhesions are still the most common cause of IO, and the rate of malignant bowel obstruction gets higher by years. Physicians can refer to these valid data to focus specifically on previous surgery history and family history of colorectal malignance. Good results could be achieved by early screen and intervention in time.

## Figures and Tables

**Table 1:** Characteristics of 4908 patients with intestinal obstruction.

	Patients
Sex	
Male/female (ratio)	2945/1963 (1.5 : 1)
Age	
<18 (*n*, %, mean ± SD)	1006 (20.50, 4.8 ± 4.4)
18-50 (*n*, %, mean ± SD)	1558 (31.74, 37.9 ± 8.8)
≥50 (*n*, %, mean ± SD)	2344 (47.76, 65.9 ± 9.2)
Occupational physical activity	1178/820/2910
Mild/moderate/severe (*n*, %)	(24.00/16.71/59.29)
Diagnostic modalities	
X-ray (*n*, %)	4908 (100.00)
CT scan (*n*, %)	2049 (42.00)
Endoscopy (*n*, %)	817 (16.65)
Etiology	
Mechanical IO (*n*, %)	4138 (84.20)
Nonmechanical IO (*n*, %)	315 (6.50)
Indeterminate IO (*n*, %)	455 (9.30)
Location of obstruction	
Small intestine (*n*, %)	3072 (62.59)
Large intestine (*n*, %)	1330 (27.10)
Indeterminate (*n*, %)	506 (10.31)
Degree of obstruction	
Incomplete (*n*, %)	2831 (57.68)
Complete (*n*, %)	2077 (42.32)
Treatment	
Conservative therapy (*n*, %)	3581 (73.00)
Surgical operation (*n*, %)	1327 (27.00)
Mortality	4.7%
Length of hospital stay (day, mean ± SD)	13.34 ± 12.79

## Data Availability

The data used to support the findings of this study are included within the article.
